# Analysis of survey on menstrual disorder among teenagers using Gaussian copula model with graphical lasso prior

**DOI:** 10.1371/journal.pone.0248340

**Published:** 2021-03-18

**Authors:** Jiali Wang, Anton H. Westveld, A. H. Welsh, Melissa Parker, Bronwyn Loong

**Affiliations:** 1 Research School of Finance, Actuarial Studies and Statistics, College of Business and Economics, The Australian National University, Canberra, ACT, Australia; 2 Canberra Endometriosis Centre, Centenary Hospital for Women and Children, ACT Health, Canberra, ACT, Australia; University of Bradford, UNITED KINGDOM

## Abstract

A high prevalence of menstrual disturbance has been reported among teenage girls, and research shows that there are delays in diagnosis of endometriosis among young girls. Using data from the Menstrual Disorder of Teenagers Survey (administered in 2005 and 2016), we propose a Gaussian copula model with graphical lasso prior to identify cohort differences in menstrual characteristics and to predict endometriosis. The model includes random effects to account for clustering by school, and we use the extended rank likelihood copula model to handle variables of mixed-type. The graphical lasso prior shrinks the elements in the precision matrix of a Gaussian distribution to encourage a sparse graphical structure, where the level of shrinkage is adaptable based on the strength of the conditional associations among questions in the survey. Applying our proposed model to the menstrual disorder data set, we found that menstrual disturbance was more pronouncedly reported over a decade, and we found some empirical differences between those girls with higher risk of developing endometriosis and the general population.

## 1 Introduction

Menstrual disturbance, which causes physical discomfort and psychological interruption, is being reported more frequently among teenage girls [1]. It may have an adverse impact on school studies, social relationships, physical and mental health [2, 3]. Early investigation of menstrual disorder symptoms may assist teenagers with early diagnosis of some severe diseases, such as endometriosis and polycystic ovarian syndrome. There is an urgent need for a better understanding of the prevalence of menstrual disturbance and variation in experiences of menstruation among teenagers.

Studies using questionnaires to investigate menstrual disorders among adolescent girls had been conducted in multiple study cohorts in different counties [2, 4–6]. They are important resources to study menstrual problems and uncover factors that cause abnormal menstrual symptoms during the pubertal development of teenage girls. The Menstrual Disorder of Teenagers (MDOT) survey was conducted in 2005 and 2016 to collect data on the menstrual pat-terns of teenage girls [7]. Both surveys were conducted in the Australian Capital Territory (ACT) using the same questionnaire. The two cohorts of participants were 15-19 years old teenage girls from 4 senior high schools in 2005 and 3 senior high schools in 2016. Consent forms were signed by all the eligible girls before participating in the surveys. The data collection was approved by ACT Health Human Research Ethics Committee (ETHLR.15.133) and the ACT Government Education Directorate. Using the collected data to conduct analysis was approved by the Human Research Ethics Committee at the Australian National University (Protocol 2017/284). The quality of the data was maximized by the careful design of the questionnaire, getting support from participating schools, and allocating time to fill in the ques-tionnaires during class. The consistency of the data from the two cohorts was guaranteed by using the same questionnaire and following the same data collection procedure from 2005 in 2016.

A sample questionnaire is attached in the S1 File. Information on the following was collected: personal information, typical menstruation characteristics, menstrual symptoms, interference with life, menstrual experiences, and knowledge and diagnosis of some menstrual diseases. Using the MDOT data set, which contains holistic information from more than 100 questions on menstruation, a range of scientific questions can be answered. In this paper, we will provide answers to the following questions using our proposed model in Section 3.

Which menstrual characteristics are changing over time? At the population level, it is of interest to note any pattern of changes of menstrual characteristics, and the MDOT survey provides a unique opportunity to answer this question.
What are the relationships between questions in the survey, i.e. typical menstrual characteristics, menstrual symptoms, and interference with life activities? Previous studies have shown that various menstrual symptoms and menstrual experiences are often interrelated with other each, and understanding how the health indicators emerge together is clinically important.
What variables are related to indicators of problematic periods, for example, pain and school absence? This regression-type analysis can reveal the relationship between a set of predictors and the responses of interest.
Can the MDOT questionnaire identify girls with a higher risk of developing endometriosis? Screening for endometriosis without relying on surgery is highly desirable among teenagers. The MDOT questionnaire covers a wide spectrum of questions and can be potentially used to develop models to evaluate individual risk.

Exploring relationships between questions is of essential importance, however there are statistical challenges to answer these clinical questions compatibly. First of all, the variables are of mixed-type (continuous, binary, ordinal), and there are constraints on their distributions. For example, the age of the senior high school participating girls was between 15 and 19; a list of statements related to periods were binary as true or false, and some life interference questions were asked using an 11-point Likert scale from 0 (no interference) to 10 (major interference). Secondly, there are a large number of questions in the questionnaire, all of which need to be analysed jointly to understand their relationships. Calculating pair-wise correlations (Pearson/Kendall’s tau/Spearman’s rho) can be misleading, because the relationship between two vari-ables may be mediated by other variables. Moreover, partial correlation which computes the linear relationship between two continuous variables after controlling for other variables is not appropriate for mixed-type variables. Furthermore, some highly correlated variables may cause multicollinearity when fitting a statistical model, making the results unstable and difficult to interpret. There are methods to reduce dimensionality via variable selection or shrink-age, for example, stepwise regression and lasso [8], but these methods do not directly account for missing values in predictors, which leads to our third challenge. There are moderate amounts of missing data in each variable, resulting in only a small proportion of girls with complete answers to every question. This is not unexpected because of the sheer number of questions in the questionnaire and the teenagers may have been unfamiliar with some terms or did not understand some questions, and so left them blank. Some missingness was caused by a misunderstanding of questions, for example, many participants answered the cycle length question which should typically lie between 21 and 45 days as the number of bleeding days, so we treated any answers outside the proper range as missing values. Fourthly, the data set is multilevel, consisting of two cohorts of girls from seven schools, which should be modeled explicitly. Lastly, there were only 7 cases of self-reported diagnosis of endometriosis in cohort 2, and that question was not collected in cohort 1. Predicting girls with higher risk of developing endometriosis from this study is challenging, given the sparse occurrence in the MDOT data set. Those questions in the MDOT data set motivate us to develop a new modeling strat-egy to handle all the challenges simultaneously.

Gaussian graphical models, also known as Gaussian Markov random fields [9], are often used to describe the non-causal relationships among variables in multivariate data sets. Consider a data set with *p* random variables *y*_1_, …, *y*_*p*_. A graphical model can be represented as *G* = (*V*, *E*), where *V* = {1, …, *p*} denote the vertices associated with the variables, and *E* âŠ‚ {(*i*, *j*) ∈ *V* × *V*: *i* < *j*} denotes the edges between a pair of variables. In a Gaussian graphical model, the random variables **y** = (*y*_1_, …, *y*_*p*_) follow a p-variate Gaussian distribution **y** ∼ *N*_*p*_(**μ**, **Ω**^**−1**^), where **μ** is the mean vector and **Ω** is the precision matrix, which is the inverse of the covariance matrix. The information on conditional independence (dependence) is embedded in the precision matrix **Ω**, where the element *ω*_*ij*_ = 0 indicates independence between variables *i* and *j* conditional on the other variables, and graphically, it corresponds to an absence of the edge between variables *i* and *j*.

However, one of the limitations of using the Gaussian graphical model is that the variables are assumed to follow Gaussian distributions. Even after some transformations, the variables can depart from Gaussianity and finding such transformations is usually impossible for discrete variables. Copula models have proven to be very powerful for modeling variables of dif-ferent types and shapes, when there is an underlying dependence among them [10]. In a copula model, the joint distribution *F* of variables *y*_1_, …, *y*_*p*_ is decomposed into the marginal distributions *F*_1_, …, *F*_*p*_ which capture the different distributions of individual variables and a copula function *C* which describes their dependence, such that *F*(*y*_1_, …, *y*_*p*_) = *C*(*F*_1_(*y*_1_), …, *F*_*p*_(*y*_*p*_)). In the Gaussian copula model, *C*(*u*_1_, …, *u*_*p*_|**Γ**) = Φ_*p*_(Φ^−1^(*u*_1_), …, Φ^−1^(*u*_*p*_)|**Γ**), where *u*_*l*_ = *F*_*l*_(*y*_*l*_), Φ_*p*_ is the cumulative distribution function of the p-variate normal distribution with mean zero and correlation matrix **Γ** (= **Ω**^**−1**^). Equivalently, the latent variable is defined as *z*_*l*_ = Φ^−1^(*F*_*l*_(*y*_*l*_)), and **z** = (*z*_1_, …, *z*_*p*_) jointly follow a Gaussian distribution **z** ∼ *N*_*p*_(**0**, **Ω**^**−1**^). Notice that with the Gaussian copula model, the statement of the conditional independence is made on the latent data **z** which are monotonically transformed from the original data **y**.

To make the Gaussian copula model generalizable to any ordered variable, we will further couple the Gaussian copula model with the extended rank likelihood method [11], so that inference on the association parameters are purely based on the rank of data without explicitly modeling the marginal distribution functions *F*_*l*_ (to be discussed in detail in Section 2.1). To handle the large number of variables in the MDOT data set, we also employ shrinkage methods to estimate a sparse Gaussian graphical model. This can be achieved by imposing a penalty on the **Ω** matrix when maximizing the log likelihood in a frequentist model [12, 13], or by putting a shrinkage prior on **Ω** in a Bayesian model [14–17].

In this paper, we propose a Gaussian copula model with graphical lasso prior to analyse the MDOT data set, to tackle all the challenges mentioned above. Built upon the extended rank likelihood Gaussian copula model [11], our model is able to capture the dependence between all the variables of mixed-type in a unified framework. The adaptive graphical lasso shrinkage prior [17] is applied to the precision matrix in the graphical model to learn a parsimonious structure of the graph. The school effects are modeled by random effects from which the cohort differences can be derived. Moreover, a predictive score of endometriosis is obtained from the posterior predictive distribution for each person. The statistical inference is carried out under a Bayesian framework where the missing data are integrated out so that the extra variability due to missingness is incorporated in the posterior. The computational algorithm is adapted from the block Gibbs sampling algorithm in the Gaussian graphical model with lasso shrinkage prior [17] and the copula model for mixed-type data in multilevel data sets [18].

The paper is structured as follows. In Section 2, we review the extended rank likelihood method and describe our proposed model. In Section 3 we apply our model to the MDOT data set, and provide our answers to the clinical research questions (a)—(d) mentioned above. Section 4 concludes the paper with some extensions. The computational algorithm is described in detail in Appendix A, and a simulation study on choosing hyper-parameter values is described in Appendix B.

## 2 Gaussian copula model with graphical lasso prior

We consider multilevel data sets, with *l* = 1, …, *p* variables, *c* = 1, …, *m* clusters and *i* = 1, …, *n*_*c*_ units within cluster *c*. Our proposed model is

$$\begin{array}{cc} & y_{icl}=F_{l}^{-1}\left(\Phi \left(z_{icl}\right)\right),\\ & p\left(z_{\text{ic}}|b_{c},\Omega \right)=N_{p}\left(b_{c},\Omega^{-1}\right),\\ & p\left(b_{c}|\Psi \right)=N_{p}\left(0,\Psi \right), \end{array}$$ (1)

where *F*_*l*_ is the distribution function of variable *y*_*l*_, the vector **z**_**ic**_ is the latent variable of unit *i* in cluster *c*, corresponding to the observed data vector **y**_**ic**_. The random effect **b**_**c**_ is a vector of length *p* for cluster *c* which follows a p-variate Gaussian distribution with unconstructed covariance matrix **Ψ**. Model (1) specifies a Gaussian copula model on the latent variables **z**, where the precision matrix **Ω** contains information of conditional independence between variables after adjustment for the cluster effects.

To obtain a sparse graphical structure, we put the adaptive graphical lasso prior on the precision matrix **Ω** as follows

$$\begin{array}{cc} & p\left(\Omega |\lambda \right)\propto \prod_{i<j}\text{DE}\left(\omega_{ij}|\lambda_{ij}\right)\prod_{i<j}^{p}\text{Exp}\left(\omega_{ii}|\lambda_{ii}/2\right)1_{\Omega \in M^{+}},i,j=1,\ldots ,p,\\ & \lambda_{ij}|s,t∼\text{Gamma}\left(s,t\right), \end{array}$$ (2)

where **λ** = {λ_*ij*_, *i*, *j* = 1, …, *p*}, and DE(⋅), Exp(⋅) and Gamma(⋅) are probability distribution functions of the double exponential, exponential and Gamma distributions respectively. The shrinkage parameter λ_*ij*_ is associated with the element *ω*_*ij*_ in the **Ω** matrix, and **Ω** is restricted to positive definite matrics **M**^+^. The two hyper-parameters *s* and *t* further control λ_*ij*_. The adaptive graphical lasso prior (2) allows for different shrinkage effects on each unique elements in **Ω**, so that it overcomes some limitations of the double exponential prior which tends to over-shrink large parameters but under-shrink small ones [19]. We will discuss the sensitivity to the choice of hyper-parameters in Section 3.5 and Appendix B. We assume a semi-conjugate Inverse Wishart prior for the covariance matrix of random effects: *p*(**Ψ**) = Inv Wishart(*ν*, **Λ**).

### 2.1 The extended rank likelihood of Gaussian copula

In the Gaussian copula model, specifying each of the marginal distributions *F*_*l*_ is labour intensive and the distributions of variables in real data sets may not be accurately represented without a large number of parameters. Some authors suggested transforming the variables using the empirical distribution ${\hat{F}}_{l}$ to obtain pseudo data and avoid the parametric estimation of marginal distributions [20]. However, this only applies to continuous variables. To link the discrete variables with continuous latent variables, Hoff provided a simple way of analysing the correlation among variables with meaningful ordering (continuous, binary and ordered categorical variables), via the extended rank likelihood [11]. This makes use of the fact that the order of the underlying latent variable is consistent with the observed data, and inference about the association parameters can be drawn from the ‘rank-based’ latent variables through a simple parametric form. Therefore, there is no need to specify the marginal distributions *F*_*l*_ directly when making inference on **Ω**, since we know *F*^−1^(Φ(⋅)) is a monotone transformation, the ordering of the data is the same as the ordering of the latent variables **z**. Specifically, observing **y** = (*y*_1_, …, *y*_*N*_) tells us that **z** = (*z*_1_, …, *z*_*N*_) must lie in the set: $\left\{z\in R^{N\times p}:\text{max}\left\{z_{hl}:y_{hl}<y_{nl}\right\}<z_{nl}<\text{min}\left\{z_{hl}:y_{hl}>y_{nl}\right\}\right\}$, where the index *n* = 1, …, *N* runs through all the observations. Let ‘*D*’ denote the set of all possible **z** which are consistent with the ordering of **y**. Then the event ‘**z** ∈ *D*’ can be treated as the observed event upon which inference of **Ω** is made, i.e. *p*(**z** ∈ *D*|**Ω**).

Computationally, any latent variables **z** associated with observed data are updated from truncated Gaussian distributions subject to their neighboring data points (see details in Step 8 in Appendix A, whereas the latent variables associated with missing data are updated from Gaussian distributions without truncations. The model is able to account for data Missing At Random (MAR), and the uncertainty due to missing values is incorporated in the posterior distribution.

### 2.2 Related works

A copula-based imputation model was proposed by fusing the extended rank likelihood for ordered variables and a multinomial probit model for nominal variables, and added random effects to the latent variables in multilevel data sets [18]. Our model (1) is the same but does not include nominal variables. As for the priors, the semi-conjugate Inverse Wishart priors were put on both **Ψ** and **Γ** = **Ω**^**−1**^ matrices in [18] so that no formal shrinkage effects through regularization priors were imposed.

For multivariate analysis with a moderate to large number of variables, there are some proposed models for learning a sparse Gaussian graphical model using shrinkage methods. This can be achieved by imposing a *L*_1_ penalty on the **Ω** matrix in the log likelihood function

$$\begin{array}{c} \text{log}\;(\text{det}\left(\Omega \right))-\text{tr}\left(\frac{S}{N}\Omega \right)-{\rho ||\Omega ||}_{1}, \end{array}$$ (3)

where **S** is the sample covariance matrix, and this optimiazation problem was solved by the interior point method in [12]. Notice that by assuming a common shrinkage parameter λ in the graphical lasso prior (2), its maximum a posteriori is the same as the maximum likelihood solution of model (3) when *ρ* = λ/*N*. The block Gibbs sampler used in our sampling algorithm follows closely the block coordinate decent method of [13] when solving (3).

Putting a different prior, the G-Wishart prior, on the precision matrix **Ω**, [16] and [14] proposed a Bayesian Gaussian copula graphical model with the extended rank likelihood, but without random effects in the model. They decomposed the posterior distribution of the graphical model as

$$\begin{array}{c} p(\Omega ,G|z\in D)\propto p(z\in D|\Omega )p(\Omega |G)p(G), \end{array}$$ (4)

with a uniform prior on the graph *G*, and a G-Wishart prior on the **Ω** matrix given a graph structure *p*(**Ω**|*G*). The G-Wishart prior is conjugate to a Gaussian graphical model that allows for the absence of some edges, which is equivalent to have some elements equal to 0 in the **Ω** matrix. When the graph is fully connected, the G-Wishart prior reduces to the Wishart prior. The difference between the graphical lasso prior in our model and the G-Wishart prior in their model is that the former has zero probability to shrink *w*_*ij*_ to 0 exactly, therefore some post processing decisions should be made to decide a graph structure. In other words, the inference of their model was done in two steps by selecting *G* and making inference on **Ω** whereas the posterior decomposition in our model was *p*(**Ω**(*G*)|**z** ∈ *D*) ∝ *p*(**z** ∈ *D*|**Ω**(*G*))*p*(**Ω**(*G*)), such that the graphical structure *G* was implied by the **Ω** matrix implicitly. [16] applied their model to a functional disability data set which contained 16 binary variables of activities of daily living, to analyse the interactions among them, whereas [21] performed an application to a dupuytren disease data set, aiming to model the relationships between the severity of the disease in the 10 fingers and potential lifestyle risk factors.

## 3 Data application to the MDOT data set

In this section we apply our model to the MDOT data set, to answer the four clinical questions listed in the introduction using the collected information from the MCMC. The data set was coded in the following way before fitting the model. We derived the variable BMI from weight and height, and the number of bleeding days from the heaviness of bleeding question. We treated the answers to the cycle length question for fewer than 21 days or longer than 45 days as missing values. As the response options available for the multiple choice questions on menstrual symptoms were not mutually exclusive (Section 3 in the MDOT questionnaire), and because we were more interested in the appearance of symptoms before and during periods, we dichotomized the choices ‘just before a period’ and ‘at the time of period’ as 1 and others as 0. For questions regarding interference with life, statements of period and knowledge of menstrual diseases, ‘do not know’ or ‘NA’ answers were treated as missing values. Finally, diagnosis of polycystic ovarian syndrome/polycystic ovaries, pelvic inflammatory disease and endometriosis were only asked in the second cohort but not in the first cohort. Nevertheless we still allowed the three questions to enter into our model by treating the answers in the first cohort as missing values, so that only answers to these three questions from cohort 2 provided information on associations with other variables.

We excluded 29 girls who answered that they did not have any periods so most of the questions were not applicable to them. The final data set contained 1039 girls from 4 schools in the first cohort and 1041 girls from 3 schools in the second cohort. Two schools participated in both cohorts, and we assumed that the intrinsic characteristics of the schools had changed over the 11-year interval; that is, we assumed responses from a school in cohort 1 were independent of responses from the same school in cohort 2. Due to these characteristics, we included 7 random effects and not 5. There were p = 105 questions entered into the Gaussian copula graphical model, including 5 continuous variables, 19 ordinal variables and 81 binary variables.

We ran the MCMC for 20,000 iterations, saving every 10^th^ scans and discarding the first half of the samples as the burn-in period. The hyper-parameters for **Ψ** were chosen to be *ν* = *p* + 2, **Λ** = **I**_**p**_, and for the shrinkage parameters λ_*ij*_ to be *s* = 10^−2^ and *t* = 10^−4^. We performed a sensitivity analysis on choosing *t* which will be described in Section 3.5. We note that the model was insensitive to the settings of other hyper-parameters. For the MCMC diagnostics, we examined the autocorrelations and traceplots of the parameters as well as checking for the mixing and convergence by the potential scale reduction factors [22] from three independent chains with different starting values. There were 4.37% and 0.348% of the parameters in the **Ω** and **Ψ** matrices with their individual potential scale reduction factors exceeding 1.05. Given the sheer number of parameters in the model, the results suggest convergence.

As an alternative approach, we also considered the Bayesian Gaussian copula graphical model of [21] who decomposed the graph as Eq (4) via the extended rank likelihood method with a G-Wishart prior. They used a trans-dimensional birth and death process to select the graph structure, by adding or removing edges in the MCMC. However their approach does not allow explicitly for adding clustering effects. They implemented their algorithm in the ‘BDgraph’ package in R [23], and we used the function ‘bdgraph’ to fit model to the MDOT data set, with 20,000 iterations, and assuming non-informative priors.

### 3.1 Cohort differences

Recall that *b*_*cl*_ denotes the random effect for school *c*Â (*c* = 1, …, 7) in question *l*Â (*l* = 1, …, 105) of the survey. Define ${\bar{b}}_{(1)l}=\frac{b_{1l}+b_{2l}+b_{3l}+b_{4l}}{4}$ to be the first cohort effect for question *l*, and similarly define ${\bar{b}}_{(2)l}=\frac{b_{5l}+b_{6l}+b_{7l}}{3}$ to be the second cohort effect. We computed the mean and the 95% highest density region of the difference $b_{dif,l}={\bar{b}}_{(1)l}-{\bar{b}}_{(2)l}$ in each question. Results are shown in the ‘GCM_Lasso 95% credible interval’ column in Table 1. If the 95% highest density region of the cohort difference *b*_*dif*,*l*_ did not contain 0, heuristically, it suggests that the cohort difference of question *l* was important. Results for the alternative method, the Gaussian copula graphical model with G-Wishart prior, are reported in the ‘BDgraph’ columns. To provide an answer to the cohort difference without modeling school effects explicitly, we added the ‘cohort’ variable as the 106^th^ variable, to indicate which cohort the respondents came from. Then we calculated the regression type coefficients of the ‘cohort’ variable on each of the other 105 variables as responses. This is similar to a regression problem in that we treated ‘cohort’ as a predictor and see if it was significantly associated with the response. The regression coefficient of the predictor ‘cohort’ on the *l*^*th*^ response variable can be derived from the covariance matrix **Γ** as $\Gamma_{106,l}\Gamma_{106,106}^{-1}$ by the conditional distribution of a multivariate Gaussian distribution, because $z_{icl}|z_{ic,106}∼N\left(b_{cl}+\Gamma_{106,l}\Gamma_{106,106}^{-1}\left(z_{ic,106}-b_{c,106}\right),\;\Gamma_{l,106}\Gamma_{106,106}^{-1}\Gamma_{106,l}\right)$, or from the precision matrix **Ω** as $-\Omega_{106,l}\Omega_{ll}^{-1}$ using the identity $\Omega_{106,l}=-\Omega_{ll}\Gamma_{106,106}^{-1}\Gamma_{106,l}$. The posterior means of the coefficients are reported in the ‘BDgraph coefficient’ column in Table 1. The estimated posterior edge inclusion probabilities between the ‘cohort’ variable and the other 105 variables are reported in the ‘BDgraph edge’ column of Table 1. When the edge inclusion probability was 0, the corresponding coefficient was 0 as well, which means that the cohort difference was insignificant.

**Table 1:** Comparison of cohort difference in each question by 1) mean responses in the two cohorts, 2) our proposed model (GCM_Lasso), 3) Gaussian copula graphical model with G-Wishart prior (BDgraph). The significant differences are in bold.

	Question	Variable name	Mean response		GCM_Lasso 95% credible interval	BDgraph edge	BDgraph coefficient
			2005	2016			
Menstrual characteristics	age	age	17.22	17.19	0.054(-0.011,0.140)	0	0.000
	BMI	BMI	21.29	21.58	-0.052(-0.126,0.030)	0	0.000
	age of first period	agefp	12.74	12.52	**0.122(0.039,0.203)**	**0.408**	-0.073
	regular period	p1q2	67%	61.6%	0.058(-0.047,0.163)	**0.201**	-0.012
	cycle length	p1q3	28.58	28.67	-0.074(-0.163,0.051)	0	0
	bleeding days	p1q4	5.96	6.21	**-0.096(-0.187,-0.022)**	**0.208**	0.013
	contain clots	p1q5	58.1%	64.7%	**-0.108(-0.197,-0.028)**	0	0.000
	school absence	p1q8	25.8%	28%	0.023(-0.079,0.117)	**0.788**	-0.094
	symptoms worsened	p1q11	26.2%	26.3%	0.07(-0.066,0.183)	0	0.000
	pain severity	p2q12	5.07	5.49	-0.072(-0.143,0.023)	0	0.000
	take medication	p2q13	65.9%	65.9%	0.093(-0.009,0.177)	0	0.000
Symptoms (before or during period)	nausea	Sec3_a	21.1%	23.5%	0.021(-0.058,0.118)	**0.782**	-0.088
	vomiting	Sec3_b	6.3%	7.2%	0.034(-0.102,0.167)	0	0.000
	bloating	Sec3_c	48.2%	46.5%	-0.021(-0.101,0.080)	0	0.000
	diarrhoea	Sec3_d	13.9%	25.8%	**-0.246(-0.360,-0.143)**	**1**	0.308
	indigestion	Sec3_e	7.7%	11%	-0.052(-0.174,0.041)	**0.472**	-0.094
	changes in appetite	Sec3_f	34.9%	40.8%	-0.074(-0.220,0.011)	0	0
	aching outside vagina	Sec3_g	17.7%	23.3%	**-0.126(-0.205,-0.013)**	0	0.000
	aching down the legs	Sec3_h	17.5%	19.6%	-0.020(-0.117,0.085)	**0.087**	-0.013
	pelvic pain aching	Sec3_i	45.9%	51.6%	-0.026(-0.104,0.093)	**0.775**	-0.026
	pelvic pain cramping	Sec3_j	52.9%	58%	-0.010(-0.118,0.064)	0	0.000
	pelvic pain stabbing	Sec3_k	27%	41.6%	**-0.203(-0.304,-0.112)**	**1**	0.219
	lower back pain	Sec3_m	36.5%	39.8%	-0.017(-0.108,0.069)	0	0.000
	pain during or after passing urine	Sec3_n	2.9%	6.5%	**-0.194(-0.310,-0.019)**	0	0.000
	pain when bladder full	Sec3_o	6.7%	10%	-0.037(-0.189,0.068)	**0.377**	-0.040
	pain before or when passing wind	Sec3_p	3.3%	6.6%	**-0.148(-0.304,-0.027)**	**0.970**	0.133
	pain when emptying bowels	Sec3_q	5.2%	8.6%	**-0.130(-0.223,-0.009)**	0	0.000
	urgent need to empty bowels	Sec3_r	6.3%	10.5%	**-0.111(-0.222,-0.023)**	0	0.000
	bleeding from bottom	Sec3_s	0.6%	2.4%	**-0.250(-0.520,-0.014)**	**0.031**	0.064
	pain during or after sexual intercourse	Sec3_t	1.4%	2.1%	-0.022(-0.188,0.114)	0	0.000
	need to pass urine often	Sec3_u	7.1%	12.6%	-0.153(-0.26,0.008)	**0.441**	0.105
	feeling really tired	Sec3_v	30.4%	29%	**0.112(0.014,0.209)**	0	0.000
	headaches	Sec3_w	19.8%	23.3%	-0.057(-0.195,0.045)	0	0.000
	thrush	Sec3_x	4.20%	8.5%	**-0.221(-0.396,-0.098)**	**0.861**	0.277
	dizziness	Sec3_y	13.1%	15.6%	-0.057(-0.153,0.050)	0	0.000
	feeling down or depressed	Sec3_z	26.7%	26.1%	0.060(-0.015,0.169)	**0.492**	-0.132
Interference with life	attending school	p3s4a	1.72	1.8	**0.092(0.023,0.189)**	0	0.000
	completing school work	p3s4b	1.91	2.47	**-0.140(-0.235,-0.047)**	**1**	0.202
	casual paid work	p3s4c	1.58	1.55	**0.105(0.016,0.192)**	**0.964**	-0.210
	social acitivities	p3s4d	2.81	3.17	-0.025(-0.107,0.046)	**0.108**	0.009
	relationship with family	p3s4e	2.33	2.45	0.041(-0.059,0.120)	**0.710**	0.102
	relationship with friends	p3s4f	1.73	1.97	-0.017(-0.090,0.064)	0	0.000
	relationship with partners	p3s4g	1.99	1.47	**0.218(0.129,0.330)**	**0.143**	-0.036
	sexual activity	p3s4h	3.69	2.46	**0.322(0.214,0.437)**	**1**	-0.169
	sport and exercise	p3s4i	3.24	4.02	**-0.141(-0.216,-0.048)**	0	0.000
	interference with lifestyle	p3s4j	1.72	1.87	**-0.163(-0.238,-0.068)**	**0.144**	0.007
	pain	p4s4k	4.59	5.15	-0.070(-0.128,0.003)	0	0.000
	heavy blood flow	p4s4l	3.48	4.48	**-0.233(-0.314,-0.147)**	0	0.000
	tiredness	p4s4m	3.88	5.06	**-0.259(-0.339,-0.175)**	**0.871**	0.279
	moods	p4s4n	4.66	5.04	-0.042(-0.130,0.023)	**1**	-0.250
	generally feeling unwell	p4s4o	3.65	4.41	**-0.108(-0.181,-0.046)**	0	0.000
	frequency of interference	p4s4r	1.79	1.95	**-0.129(-0.202,-0.049)**	**0.147**	0.006
Problematic menstruation	I usually have a period every month	p4s5a	87.2%	86.8%	0.096(-0.011,0.232)	0	0.000
	I have never missed a period	p4s5b	44.4%	42.8%	-0.012(-0.151,0.093)	0	0.000
	I have had problems with my periods	p4s5c	41.3%	44.8%	0.010(-0.078,0.128)	**0.214**	0.001
	My periods seem pretty normal	p4s5d	81.8%	79.6%	0.052(-0.040,0.148)	**0.577**	-0.016
	I have had tests because things weren’t right with my period	p4s5e	11.8%	12.8%	0.011(-0.092,0.152)	0	0.000
	I have a period problem that has a name	p4s5f	3.9%	4.3%	-0.025(-0.204,0.131)	0	0.000
	I am on the pill	p4s5g	23.2%	21.8%	-0.025(-0.120,0.059)	**0.083**	-0.002
	I take the pill to regulate my periods	p4s5h	18.1%	19.1%	-0.075(-0.162,0.035)	0	0.000
	I take the pill to prevent pregnancy	p4s5i	20.5%	15.3%	0.090(-0.011,0.189)	0	0.000
	I take the pill to help period pain	p4s5j	16.2%	18.1%	-0.045(-0.169,0.064)	**0.362**	0.011
	I have never taken the pill	p4s5k	65.1%	66%	-0.007(-0.095,0.073)	**0.182**	0.005
	Periods don’t worry me too much	p4s5l	73.2%	64.1%	**0.114(0.006,0.193)**	**0.193**	-0.008
	Periods worry me a lot	p4s5m	17.8%	24.3%	-0.115(-0.228,0.010)	**0.024**	0.004
	I think my periods are normal most of the time	p5s5a	86.4%	84.8%	0.033(-0.062,0.127)	0	0.000
	I sometimes think there is something wrong with my periods	p5s5b	29%	36.5%	-0.096(-0.193,0.016)	**0.154**	0.095
	I am sure there is something wrong with my periods	p5s5c	10.6%	11.7%	0.017(-0.126,0.107)	0	0.000
	I have never used a tampon	p5s5d	30.3%	38.8%	**-0.158(-0.261,-0.072)**	**0.287**	0.007
	I have tried to used a tampon but can’t get it in	p5s5e	9.6%	14.8%	**-0.147(-0.266,-0.023)**	0	0.000
	I am not interested in using a tampon	p5s5f	24%	38.5%	**-0.292(-0.371,-0.174)**	**0.079**	0.133
	I can insert a tampon but it is too uncomfortable	p5s5g	10.9%	19%	**-0.231(-0.346,-0.074)**	0	0.000
	I only use tampons	p5s5h	16.4%	11.4%	**0.130(0.016,0.251)**	**0.445**	-0.020
	I only use pads	p5s5i	37.5%	51.1%	**-0.205(-0.330,-0.098)**	**1**	0.031
	I use pads and tampons	p5s5j	57.5%	47.4%	**0.137(0.047,0.251)**	0	0.000
	I often get lots of pimples on my face	p5s5k	63.4%	75.5%	**-0.197(-0.287,-0.074)**	**0.266**	0.028
	I often get lots of pimples on my back	p5s5l	31.3%	37.4%	-0.079(-0.208,0.017)	0	0.000
	I often get lots of pimples on my chest	p5s5m	29.6%	30.5%	**-0.208(-0.331,-0.068)**	**0.825**	0.034
	I have more hair than usual growing on my body	p5s5n	8.4%	15.4%	**-0.209(-0.326,-0.063)**	**0.130**	0.094
	I have had a blood test for my period pain	p5s5o	4.1%	6.1%	-0.085(-0.251,0.047)	0	0.000
	I have had an ultrasound to look for causes of my pain	p5s5p	5.8%	8.1%	-0.071(-0.203,0.051)	**0.136**	0.003
	I have had an operation to look for causes of my pain	p5s5q	0.9%	1.5%	-0.054(-0.255,0.201)	0	0.000
	I talk to my friends about my periods	p5s5r	71%	74.9%	-0.091(-0.197,0.015)	0	0.000
	I talk to someone in my family about my periods	p5s5s	65.2%	74.3%	**-0.180(-0.297,-0.081)**	**1**	0.255
	I have talked to my teacher/school counselor about my periods	p5s5t	2%	4.6%	**-0.210(-0.425,-0.033)**	0	0.000
	I have talked to my GP about my periods	p5s5u	33.8%	32%	**0.105(0.012,0.208)**	0	0.000
	I have talked to a specialist doctor about my periods	p5s5v	8.7%	10.9%	-0.064(-0.208,0.055)	**0.192**	0.010
	I have talked to a naturopath about my periods	p5s5w	6%	3.3%	**0.192(0.037,0.355)**	**1**	-0.298
	I am grumpy before or during my periods	p5s5x	77.7%	82.2%	-0.063(-0.155,0.034)	0	0.000
	I am grumpy all the time	p5s5y	10.3%	23.4%	**-0.374(-0.505,-0.273)**	**1**	0.603
	I get teary before or during a period	p5s5z	54.8%	62.4%	**-0.080(-0.152,-0.005)**	**1**	0.137
	I feel overwhelmed before or during a period	p5s5aa	34%	49.4%	**-0.257(-0.340,-0.144)**	**0.804**	0.221
	I often want to withdraw or hide when I have my period	p5s5bb	27.5%	48.8%	**-0.355(-0.478,-0.260)**	**1**	0.379
	My periods don’t affect my moods	p5s5cc	21%	15.8%	0.095(-0.003,0.229)	**0.100**	-0.009
Menstrual diseases	have heard of PCOS	p6s7a	19%	29.4%	**-0.320(-0.412,-0.227)**	**1**	0.388
	have heard of Endometriosis	p6s7b	23.2%	31.3%	**-0.279(-0.356,-0.182)**	0	0.000
	have heard of PID	p6s7c	30.9%	24.7%	-0.083(-0.195,0.009)	**0.264**	-0.032
	mother or sisters have period problems	p6s7d	29.5%	32.5%	-0.033(-0.151,0.051)	0	0.000
	mother or sisters have severe period pain	p6s7e	36.1%	39.4%	-0.015(-0.108,0.117)	0	0.000
	mother or sisters have PID	p6s7f	0.7%	1.4%	-0.073(-0.332,0.192)	**1**	0.308
	mother or sisters have PCOS	p6s7g	2.8%	5.2%	-0.149(-0.352,0.079)	**0.196**	0.015
	mother or sisters have endometriosis	p6s7h	1.7%	4.2%	-0.168(-0.316,0.014)	0	0.000
	diagnosed with PCOS	p6s7i	NA	2.1%	0.025(-0.123,0.195)	0	0.000
	diagnosed with PID	p6s7j	NA	0.5%	0.023(-0.174,0.236)	0	0.000
	diagnosed with endometriosis	p6s7k	NA	0.6%	0.100(-0.178,0.457)	**0.198**	0.004

The number of significant differences implied by our proposed model and the ‘BDgraph’ method were 45 and 55 respectively. Many questions reached the same conclusion by both methods. For example, 27% of girls suffered from stabbing pelvic pain in 2005, and the number rose to 41.6% in 2016. The 95% credible interval of $b_{dif,l}={\bar{b}}_{(1)l}-{\bar{b}}_{(2)l}$ was (-0.304,-0.112), which means that the mean random effect for cohort 1 was significantly lower than cohort 2, suggesting more girls in 2016 reported a positive answer to this question. In the ‘BDgraph’ method, the edge inclusion probability of the ‘cohort’ variable and the ‘stabbing pelvic pain’ question was 1, and the coefficient was 0.219, indicating a higher likelihood of having this symptom in the second cohort. Both models provided evidence that menstrual disturbance was generally getting worse or more reported over the decade. More girls suffered from diarrhoea, pain before or when passing wind, bleeding from the bottom, and thrush before or during their periods. More girls also reported higher interference with life (completing school work, feeling tired), increased worry about their periods, and more pimples. Sanitary use habits also changed, as girls were more likely to use pads than tampons. Moreover, the psychological related questions indicated a higher level of stress and depression, as 23.4% felt grumpy all the time and 62.4% got teary before or during a period in the second cohort compared with 10.3% and 54.8% respectively in the first cohort, and almost a half of the girls felt overwhelmed (49.4%) and wanted to withdraw or hide (48.8%) before or during their period in 2016, increasing by 15.4% and 21.3% respectively compared with 11 years ago. The two Bayesian approaches further confirmed these significant cohort differences. The score of interference of sexual activity dropped significantly from 3.69 to 2.46 (on a 11-point Likert scale), which was evidenced by the fact that the credible interval was above 0 (0.214,0.437) in our proposed model and the coefficient was negative (-0.169) with edge inclusion probability equal to 1 in the ‘BDgraph’ method. Some population characteristics were quite consistent though, as the average age was around 17.2 and BMI was around 21.4 in both cohorts. However some of the menstrual characteristics had changed significantly, for example, the age of menarche decreased by 3 months and bleeding time increased by 0.25 days on average. The earlier age at puberty may be explained by socio-economic changes and better diet, among other reasons. Overall our results demonstrate a worsening in symptoms and an urgent need to investigate menstrual problems among teenage girls, through various channels like GP consultations, school and family education. However, we acknowledge that the differences between cohorts could be the result of the advancement in medical technology, enhancement in knowledge, as well as more self-awareness. Further investigations that control for mediating factors are needed.

### 3.2 Conditional associations among variables of MDOT

**Fig 1:**
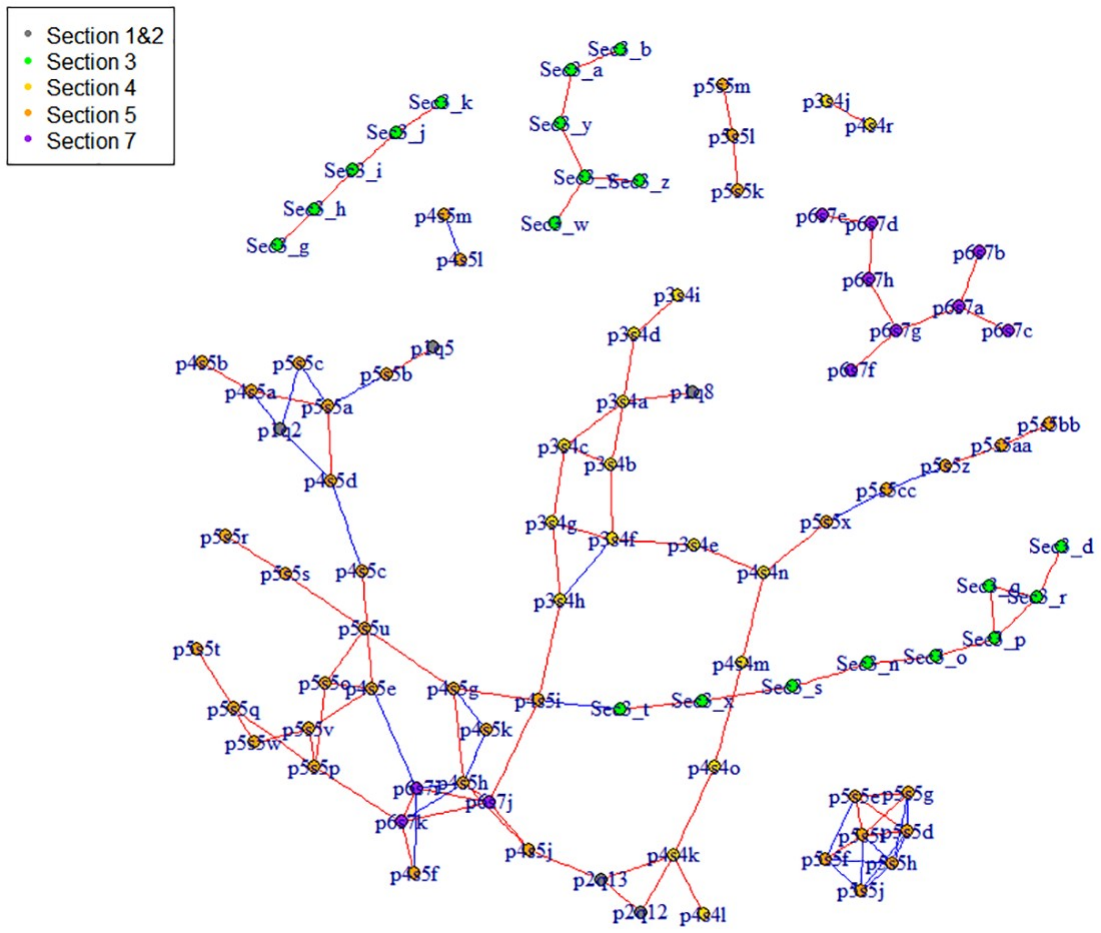
Graphic representation of conditional dependence among the questions in the MDOT data set. Edges in red denote positive relationships and blue denote negative relationships. Questions from different sections in the questionnaire are plotted with different colors.

Graphical models are a powerful tool to describe associations among variables. In our proposed graphical model (1), each element in the **Ω** matrix describes the association between a pair of variables on the latent scale, conditional on the other variables and the school effects. That is, it depicts the genuine associations between two variables after controlling for any known source of information. As the graphical lasso prior places zero probability on the absence of edges, we applied a post-processing procedure after running the MCMC to select those edges with stronger associations. In the application to the MDOT data set, we plotted the top 3% of the parameters in the upper diagonal matrix of **Ω** based on the means for each marginal posterior distribution. Note that we only plot 3% edges for a simple visualization of the most important edges, nevertheless the conditional associations between each pair of variables were still encoded in the precision matrix from the MCMC samples. In Fig 1, we removed those isolated questions, and marked the questions from different sections using different colors. Positive relationships were denoted by red edges and negative relationships by blue. The graph was plotted using the ‘igraph’ package in R [24].

The data entry coding of variables along with the actual questions in the questionnaire were listed as ‘variable name’ and ‘question’ respectively in Table 1. The variable coding rules generally follow a ‘page(p)section(s)question(q)’ format. For example, the variable ‘p3s4a’ represents the first question (a) of section 4 in page 3 in the MDOT survey, which is ‘periods have interfered with attending school’. Because we recoded the data in section 3 of the questionnaire, those questions were denoted as ‘Sec3_’ from ‘a’ to ‘z’.

We empirically grouped the 105 variables into several clusters in Fig 1. Overall, the segmentation was reasonably clear, although some clusters appeared to merge with each other. Questions from the same section of the questionnaire tended to cluster together, because they asked about similar domains of menstruation and girls may provide more consistent answers to questions closer to each other in the survey. It is also interesting to see that some questions that were not asked in a consecutive order or were far apart in the questionnaire were still clustered together. For example, the period regularity and clots questions on page 1 sat together with a list of girls’ perception questions of their periods on pages 4 and 5. Based on Fig 1, we grouped the non-isolated variables into the following clusters with a communal name in each cluster.

- Premenstrual symptoms: nausea (Sec3a), vomiting (Sec3b), feeling really tired (Sec3v), headache (Sec3w), dizziness (Sec3y), feeling down or depressed (Sec3z).
- Knowledge of menstrual diseases: have heard of polycystic ovarian syndrome/polycystic ovaries (PCOS) (p6s7a); endometriosis (p6s7b); pelvic inflammatory disease (PID) (p6s7c), mother or sister have period problems (p6s7d); severe period pain (p6s7e); PID (p6s7f); PCOS (p6s7g); endometriosis (p6s7h).
- Pimples: get lots of pimples on my face (p5s5k); back (p5s5l); chest (p5s5m).
- Atypical bowel and urinary symptoms: diarrhea (sec3d), pain during or after passing urine (sec3n), pain when bladder full (sec3o), pain before or when passing wind (sec3p), pain when emptying bowels (sec3q), urgent need to empty bowels (sec3r), bleeding from bottom (sec3s), pain during or after sexual intercourse (sec3t), thrush (sec3x) before or during period.
- Aching: aching outside vagina (sec3g), aching down the legs (sec3h), pevic pain aching (sec3i); cramping (sec3j); stabbing (sec3k) before or during period.
- Sanitary use: never used a tampon (p5s5d), tried to use a tampon but cannot get it in (p5s5e), not interested in using a tampon (p5s5f), can insert a tampon but too uncomfortable (p5s5g), only use tampon (p5s5h), only use pad (p5s5i), use pad and tampon (p5s5j).
- Moods: tiredness/fatigue (p4s4m); moods (p4s4n); generally feeling unwell (p4s4o), grumpy (p5s5x); teary (p5s5z); overwhelmed (p5s5aa); want to withdraw or hide (p5s5bb); do not affect my moods (p5s5cc) before or during period.
- Perception: regular period (p1q2), clots (p1q5), usually have period every month (p4s5a), never missed a period (p4s5b), have problems with my periods (p4s5c), my periods seem pretty normal (p4s5d), my period are normal most of the time (p5s5a), sometimes think there is something wrong with my periods (p5s5b); sure there is something wrong with my periods (p5s5c).
- Consultation and test: had tests (p4s5e), blood test (p5s5o), ultrasound (p5s5p), operation (p5s5q), talked to friends (p5s5r); someone in my family (p5s5s); teacher/school counselor (p5s5t); GP (p5s5u); specialist doctor (p5s5v), naturopath/herbalist/acupuncturist (p5s5w) about my periods.
- Interference: school absence (p1q8), attending school (p3s4a), completing school work (p3s4b), casual paid work (p3s4c), social activities (p3s4d), relationship with family (p3s4e); friends (p3s4f); partners (p3s4g), sexual activity (p3s4h), sport and exercise (p3s4i).
- Medication and menstrual diseases: pain severity (p2q12), take medication (p2q13), interference pain (p4s4k), heavy blood flow (p4s4l), had a period problem that has a name (p4s5f), on the pill (p4s5g), take pill to regulate periods (p4s5h), take pill to prevent pregnancy (p4s5i), take pill to help period pain (p4s5j), never take pill (p4s5k), diagnosed with PCOS (p6s7i); PID (p6s7j); endometriosis (p6s7k).

Some variables had relatively weaker conditional relationships with all the other variables, so they formed their own isolated clusters. They included some menstrual characteristics: total bleeding days, cycle length, age of menarche, and personal characteristics: age and BMI.

Knowing the grouping of variables can help clinicians better understand the interactions among menstrual symptoms and menstrual experiences. Another potential use is to reduce the length of the questionnaire by condensing the questions from one cluster into a single question. At the time the MDOT survey was conducted, it would take at least 20 minutes to complete the 6-page questionnaire. Fortunately the participating schools allocated school time for girls to finish their questionnaires so that the completion rates were quite high, otherwise the quality of a lengthy questionnaire cannot be guaranteed. It would be beneficial to design a shorter version of the MDOT questionnaire, without losing important information.

### 3.3 Conditional associations with pain severity

It is reported that 93% of adolescents experienced pain during menstruation [7] and pain is treated as a typical menstrual disturbance indicator. In the MDOT questionnaire, the pain severity variable (p2q12), was measured on an 11-point Likert scale, and we would like to know which variables in the questionnaire were associated with pain. In this case, the latent variable associated with pain severity was treated as the response, and we examined the conditional associations of the other 104 predictors with the response. Specifically, if the pain severity variable was the *l*^*th*^ variable, the coefficients of the other variables (a vector of length 104) on pain were $\Gamma_{-l,-l}^{-1}\Gamma_{-l,l}$ (similar to the formula showed in Section 3.1), and the 95% credible intervals were obtained from the highest density regions in the MCMC samples.

Table 2 shows the variables associated with pain severity whose 95% credible intervals of the conditional association parameters did not contain 0. Not surprisingly, only 9 variables were found to be significantly related to pain variable. This may be due to controlling for a large number of variables but also the shrinkage effect induced by the graphical lasso prior. It is found that a worsening of symptoms over the past 12 months (p1q11), taking medication (p2q13), having pelvic pain aching (Sec3i), scoring higher on interference with life because of pain (p4s4k), more frequent interference with life (p4s4r), never missing a period (p4s5b), having problems with periods (p4s5c), having an ultrasound to look for causes of pain (p5s5p), and getting teary before or during a period (p5s5z) were related to a higher score of pain. The conditional associations of other key variables, for example, school absence, can be analysed similarly.

**Table 2:** The conditional association parameters of variables related to high pain score whose 95% credible intervals do not contain 0.

Variables	95% credible intervals
symptoms worsened (yes 1, no 0)	0.110(0.057,0.169)
take medication (yes 1, no 0)	0.219(0.157,0.298)
pelvic pain aching (yes 1, no 0)	0.061(0.005,0.136)
interference pain (no interference 0—major interference 10)	0.444(0.370,0.518)
frequency of interference (some periods 1, most periods 2, all periods 3)	0.031(0.002,0.076)
never missed a period (yes 1, no 0)	0.052(0.004,0.082)
had problems with my periods (yes 1, no 0)	0.061(0.002,0.134)
had an ultrasound (yes 1, no 0)	0.072(0.003,0.178)
get teary before or during a period (yes 1, no 0)	0.038(0.010,0.084)

### 3.4 Prediction of endometriosis

Another useful application of our proposed model is for prediction of endometriosis using the posterior predictive distribution. This is a very challenging task because the disease is rarely diagnosed among teenagers, resulting in a very small number of self-reported cases of endometriosis in the MDOT data set. However, research has found that the disease is not that rare among teenage girls and there is a misconception that girls at a younger age are unlikely to develop the disease so that they are seldom referred to specialists (less than 10% in our study population) or undergo an operation (1%) to look for causes of abnormal menstrual symptoms. It is reported that there have been up to 9 years delay in diagnosis of endometriosis among teenagers compared with women aged > 30 [25, 26]. Therefore, early intervention of endometriosis is crucial for general well-being, to minimize disruption of life activities and reduce adverse effects on fertility. Clinicians could potentially follow up with those girls who show a higher risk of developing endometriosis.

In our data set, the question about diagnosis of endometriosis (p6s7k) was only asked in the second cohort where there were 7 cases reported out of 1041 girls. Among the 7 cases we excluded one girl as she stated that she did not have any periods, so she was not considered as part of our study population. We computed a predictive score of endometriosis for each person from the posterior predictive samples. We used the variable index *e* to denote the endometriosis variable, and the steps to derive the predictive score $y_{i,c,e}^{*}$ for girl *i* in school *c* are described as follows:

1. Sample an index *s* from the saved MCMC samples;
2. Extract the latent variables and parameters **z**^**s**^, **b**^**s**^, **Γ**^**s**^ with index *s*;
3. For each individual, sample a latent endometriosis variable from $z_{ice}^{s,*}∼N\left(b_{ce}^{s}+\left(z_{\text{ic},-e}^{s}-b_{c,-e}^{s}\right)\Gamma_{e,-e}^{s}{\left(\Gamma_{-e,-e}^{s}\right)}^{-1},\;\Gamma_{ee}^{s}-\Gamma_{e,-e}^{s}{\left(\Gamma_{-e,-e}^{s}\right)}^{-1}\Gamma_{-e,e}^{s}\right)$;
4. Determine the binary endometriosis variable $y_{ice}^{s,*}=\{\begin{array}{c} 1\;\;\text{if}\;z_{ice}^{s,*}>\text{min}\left\{z_{ice}^{s}|y_{ice}=1\right\},\\ 0\;\text{otherwise} \end{array}$
5. Repeat steps 1-4 500 times.

In step 3 above, the formula to calculate the predictive latent variable $z_{ice}^{s,*}$ is the same as step 8 of the computational algorithm in Appendix A, but without truncation. This is because when drawing posterior predictive samples, we treated the endometriosis variable as ‘missing’ so that $z_{ice}^{s,*}$ did not have to be constrained by its neighboring *y* values. In step 4 above, we compared the posterior predictive samples $z_{ice}^{s,*}$ with those $z_{ice}^{s}$ from the MCMC samples whose corresponding *y*_*ice*_ = 1. The smallest $z_{ice}^{s}|y_{ice}=1$ among the 6 girls was used as the benchmark to determine if $y_{ice}^{s,*}$ equaled 1 or 0. The sampling algorithm for the extended rank likelihood method ensures that the latent variable $z_{ice}^{s}$ of the diagnosed girls must be greater than those who were not diagnosed. If the predictive latent variable $z_{ice}^{s,*}$ exceeded the benchmark, then we set the predictive endometriosis variables $y_{ice}^{s,*}=1$, and 0 otherwise. Steps 1-4 were repeated 500 times to produce the posterior predictive samples of the binary variables $y_{ice}^{s,*},s=1,\ldots ,500$ for each girl, and the predictive probability of endometriosis ${\bar{y}}_{ice}^{*}$ was taken to be the average value of those $y_{ice}^{s,*}$.

**Fig 2:**
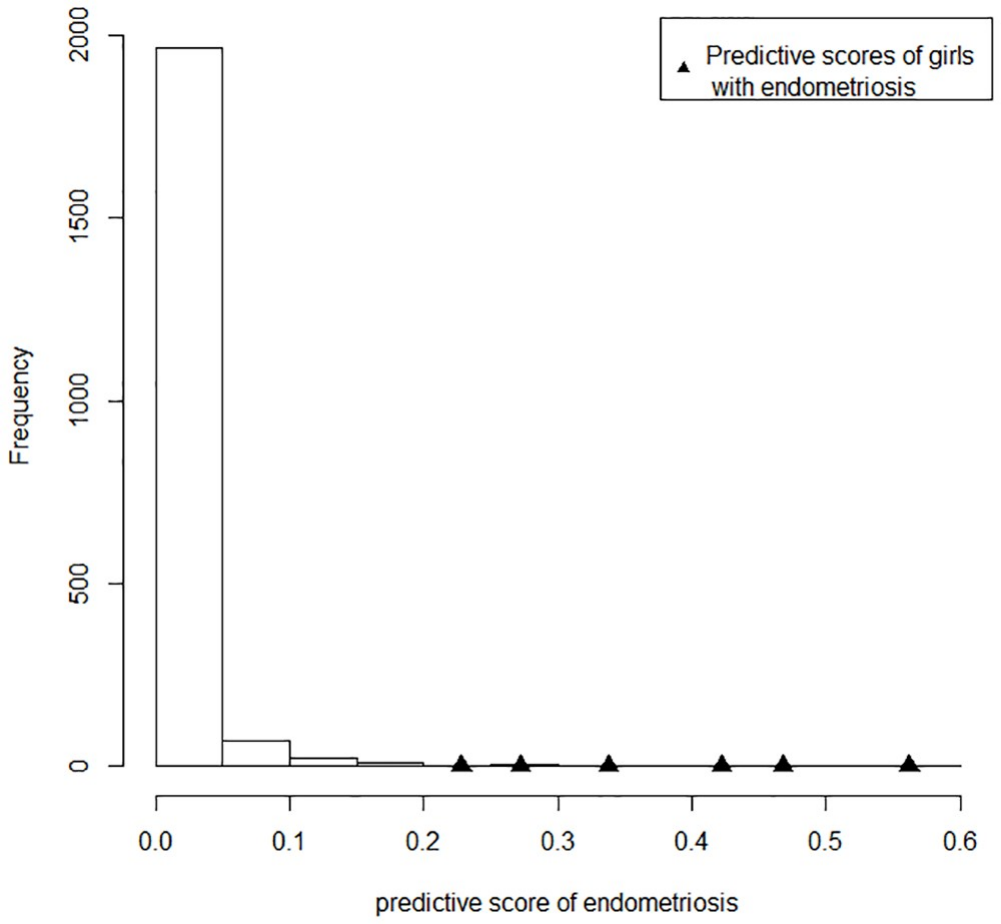
Histogram of the predictive scores of endometriosis from posterior predictive samples for each person. The scores for the 6 girls with endometriosis were marked in solid triangles.

To see if our model was a good fit to the data, and if our predictive scores were able to reflect the self-reported diagnosis of endometriosis, we examined the predictive scores of endometriosis ${\bar{y}}_{ice}^{*}$ for each girl in our study population, along with the predictive scores for the 6 girls with endometriosis marked in solid triangles in Fig 2. The 6 scores were 0.228,0.468,0.272,0.338,0.562,0.422 respectively. They indeed scored higher than the majority of individuals, suggesting reasonable predictive performance of our model. We further compared the conditional distributions in some questions of the girls with higher predictive scores in the top 10% quantile (in total 208 girls) with unconditional distributions in our study population (that is, everyone in the sample). We selected those questions that were indicative of menstrual disturbance and possible endometriosis or were expected to show differences between girls who bear higher risk of endometriosis and the general population. The results are summarized in Table 3. Firstly, 65% of girls with higher scores suffered from severe pain (scored 7-10 in the 11-point pain severity variable) which was about the same percentage who had mild pain (scored 0-6) in the general population. Only 2% of girls missed school for every period in the whole population but the number was predicted as high as 8.3% in the girls with higher scores, and nearly half of them had experienced school absence because of their periods. About two fifths of girls (40.9%) with higher predictive scores reported they had regular periods compared with two thirds (64.3%) in the whole sample. In addition, two fifths (38.7%) believed there was something wrong with their periods in the higher risk girls compared with merely 11.2% as a whole. Finally, more girls with higher scores had sought advice from their GP or specialist, or had blood tests, an ultrasound or operation to look for causes of their period pain than the general population.

**Table 3:** Comparisons of distributions of selected variables between the girls with predictive scores for endometriosis in the top 10% quantile and the study population.

		Girls with higher disturbance scoresw	Study population
Pain severity	mild pain	35%	61.4%
	severe pain	65%	38.6%
School absence	no	51.1%	73.1%
	some periods	40.6%	24.9%
	every period	8.3%	2%
Regular period	yes	40.9%	64.3%
	no	59.1%	35.7%
Sure something wrong	yes	38.7%	11.2%
	no	61.3%	88.8%
talked to GP	yes	62.1%	32.9%
	no	37.9%	67.1%
talked to Specialist	yes	29.7%	9.8%
	no	70.3%	90.2%
Blood test	yes	22.5%	5.1%
	no	67.5%	94.9%
Ultrasound	yes	29.3%	6.9%
	no	70.7%	93.1%
Operation	yes	3.3%	1.2%
	no	96.7%	98.8%

Here we proposed a way to predict endometriosis using the MDOT questionnaire, and the above analyses indicates some evidence of success for predicting endometriosis and to show differences between those girls with a higher risk of developing endometriosis and the study population. Practically, to predict the likelihood of developing endometriosis for a new girl, we can ask her to fill in the MDOT questionnaire and include her in the model by treating the endometriosis question as a ‘missing value’, and then use the procedure described above to compute her predictive score. This is essentially what has been achieved in our application to the MDOT data set to predict the probability of having endometriosis for girls in cohort 1 without their answers to that question. However, we need to be very cautious of any conclusions made, because the small number of positive cases in the data set may lead to large uncertainty in the predictive score. Moreover, a self-reported diagnosis need not necessarily be the same as a pathological diagnosis, especially when only 23.2% and 31.3% of girls had heard of endometriosis in 2005 and 2016 respectively.

### 3.5 Sensitivity analysis on the MDOT data set

In this section we discuss the issue of choosing hyper-parameters *s* and *t* which control the shrinkage parameter λ_*ij*_. In model (1), we put semi-conjugate priors on the covariance matrix **Ψ** of the random effects, and the hyperparameters were chosen to be weakly informative as *ν* = *p* + 2 and **Λ** = **I**_**p**_. For the adaptive graphical lasso prior (2) of the precision matrix **Ω**, the choice of the shrinkage parameter λ_*ij*_ is adapted from *ω*_*ij*_, and is also controlled by the hyper-parameters *s* and *t*. From step 4 of the sampling algorithm in Appendix A, the fully conditional distribution of λ_*ij*_ followed a gamma distribution: λ_*ij*_ ∼ Gamma(shape = 1 + *s*, rate = |*ω*_*ij*_| + *t*), so that the conditional mean of λ_*ij*_ is $\frac{1+s}{|\omega_{ij}|+t}$. A larger value of *t* leads to a smaller λ_*ij*_, thus penalizing *ω*_*ij*_ less, whereas decreasing *t* towards 0 will push the penalty towards infinity. [17] showed that the hyper-parameter *t* needs to be chosen small relative to |*ω*_*ij*_| to appreciate the adaptiveness. The shrinkage effects of varying the hyper-parameter *t* were demonstrated in a simulation experiment in Appendix B.

With the real data application on the MDOT data set, we set *s* = 0.01 to represent a weak prior degrees of freedom of each individual *ω*_*ij*_, and conducted a sensitivity analysis by choosing between three t values: *t* = 10^−4^, 10^−5^, 10^−6^. The results of cohort differences (Section 3.1) and conditional associations with pain severity (Section 3.3) did not change much. However, the graphs with top 3% of the edges plotted (Section 3.2) varied slightly. For example, some clusters merged or split, and some variables entered or dropped out in the graph compared with Fig 1. We also examined the posterior predictive scores for endometriosis as in Section 3.4, and results showed that the scores of 6 and 5 self-reported cases (out of 6) sat above the 90% quantile in the study population when *t* = 10^−5^ and 10^−6^ respectively. Overall speaking, the model was not very sensitive to the *t* values within a moderate range. The model with *t* = 10^−4^ achieved an adequate amount of shrinkage and good performance for the prediction of endometriosis, whereas setting *t* = 10^−6^ seemed to over shrink the conditional associations and setting *t* > 10^−4^ did not fully resolve the multicollinearity problem. Therefore we chose to report the results from the model with *t* = 10^−4^ in Sections 3.1–3.4.

## 4 Conclusions

Motivated by the MDOT data set, we proposed a Gaussian copula model with graphical lasso prior to learn the conditional independence (dependence) among a large number of variables. The copula model with the extended rank likelihood is able to accommodate ordered variables in the joint model by mapping the observed data onto latent variables which are assumed to follow a multivariate Gaussian distribution. Due to the relatively large number of questions and strong associations between some variables, we put a shrinkage prior on the precision matrix for the latent variables, to encourage sparsity in the conditional associations. The approach is implemented in an R package called “GCMlasso”, which is available from github.com/jialiwang1211/GCMlasso.

The school effects are conveniently modeled by the random effects from which the cohort difference can be derived. This approach can be used with similar repeated cross-sectional survey data where the same questions are asked to different sample of individuals at each time and the questions are then compared over time.

Compared with the G-Wishart prior, the graphical lasso prior performs graphical structure selection and precision matrix estimation simultaneously, although some post-processing decisions are needed to determine the ‘significant’ edges. With the graphical lasso prior, we can obtain the conditional associations between all the questions, and look at the associations between any two variables no matter how big or small. When applied to the MDOT data set, we used an ad-hoc approach by selecting the top 3% of largest elements in the precision matrix to draw a graph. We can vary the quantile level to plot a sparser or denser graph, nevertheless the full information of conditional independence is encoded in the precision matrices from MCMC.

In our application to the MDOT data set, we treated those ‘do not know’, ‘NA’ answers and the blank cells as missing data, and assumed they were missing at random. However in some questions, ‘NA’ may mean a negative answer, or another category. For example, an ‘NA’ answer to the True or False question ‘I have had a blood test for my period pain’ was more likely to mean having no tests; an ‘NA’ answer to the Yes or No question ‘have your period symptoms worsened over the past 12 months’ probably neither means worsened nor improved, but having no period symptoms at all. An extension of our model would be treating ‘NA’ as a separate category, and use a multinominal probit model for nominal variables [27]. With shrinkage methods, it is desirable to include or exclude the entire nominal variable instead of selecting an individual category. This can be achieved by using methods similar to group lasso where the penalty is applied to a matrix norm of each nominal variable [28, 29]. Moreover, some questions were regarded as sensitive, such as sexual activities and family history diseases, and the missing at random assumption can be violated because the probability of missing a record may depend on the record value itself. If the missingness was assumed to be not missing at random, some extra modeling efforts of the missing process will be needed.

Using the MDOT data set, we showed how our model was fitted in a single MCMC run but could be used for answering a range of research questions by extracting information from the MCMC. We found that menstrual disturbance was more pronouncedly reported over a decade and some menstrual patterns were shifted dramatically, therefore there is an urgent need to investigate the pathological reasons for the changes. Furthermore, some questions were strongly correlated even after controlling for other information, and it is worthwhile looking at the interactions among a group of variables instead of individual questions that lead to some menstrual symptoms. Lastly, our prediction of endometriosis showed that there were empirical differences between girls with higher risk of endometriosis and the general study population. Developing a non-invasive screening tool for predicting endometriosis is of clinical interest.

## 5 Appendix

### 5.1 Appendix A: Sampling algorithm

The sampling algorithm is adapted directly from [17], who proposed a block Gibbs sampler by data augmentation to update the *Ω* matrix column-wise with the graphical Lasso prior, and from [18] which provided the formulas to sample from the extended rank likelihood Gaussian copula model with random effects. The steps of the algorithm for our proposed model (1) are outlined below.

1. Sample the random effects **b**_**c**_, *c* = 1, …, *m*
$b_{c}∼N\left({\left(\Psi^{-1}+n_{c}\Omega \right)}^{-1}\Omega \sum_{i=1}^{n_{c}}z_{i},{\left(\Psi^{-1}+n_{c}\Omega \right)}^{-1}\right)$, where *n*_*c*_ is the number of observations in cluster *c*;
2. Sample the covariance matrix $\tilde{\Psi }$ for the random effects
$\tilde{\Psi }∼\text{Inverse}\;\text{Wishart}\left(\nu +m,\Lambda +\sum_{c=1}^{m}b_{c}b_{c}^{T}\right)$;
3. Partition the precision matrix **Ω**, scaling matrix **Υ**, and empirical covariance matrix **S**, and move each column of these matrices to the last column in turn, to facilitate updating them column-wise. For example, if **Ω** is a *p* × *p* matrix, then **Ω**_**11**_ is a (*p* − 1) × (*p* − 1) matrix, **ω**_**12**_ and $\omega_{21}^{T}$ are column vectors of length *p* − 1, and *ω*_22_ is a scalar. Elements in the matrices **S** and **Υ** are defined similarly. The scaling matrix **Υ** is introduced as an auxiliary variable to facilitate sampling the precision matrix in step 6.
$\Omega =(\begin{array}{cc} \Omega_{11} & \omega_{12}\\ \omega_{21} & \omega_{22} \end{array})$, $S=(\begin{array}{cc} S_{11} & s_{12}\\ s_{21} & s_{22} \end{array})$, $ϒ=(\begin{array}{cc} {ϒ}_{11} & \tau_{12}\\ \tau_{21} & 0 \end{array})$, where *Υ* is a symmetric matrix with zeros along the diagonal, and **S** = (**z**_**ic**_ − **b**_**c**_)(**z**_**ic**_ − **b**_**c**_)^*T*^;
4. Sample the shrinkage parameters λ_*ij*_, *i*, *j* = 1, …, *p*
  λ_*ij*_ ∼ Gamma(shape = 1 + *s*, rate = |*ω*_*ij*_| + *t*);
5. Sample the elements *τ*_*ij*_ in the scaling matrix **Υ**, *i*, *j* = 1, …, *p*
$\tau_{ij}∼1/\text{Inverse}\;\text{Gaussian}\left(\lambda_{ij}/\omega_{ij},\lambda_{ij}^{2}\right)$;
6. Sample the **Ω** matrix column-wise using a data augmentation algorithm. This step is repeated *p* times by rearranging each column to be the last column in the matrices in step 3 in turn, and sample the parameters. The parameter λ_22_ is defined as the shrinkage parameter associated with *ω*_22_.
  Sample the auxiliary variables *γ* ∼ Gamma(shape = *N*/2 + 1, rate = (*s*_22_ + λ_22_)/2), ***β*** ∼ *N*(−**Cs**_**12**_, **C**), where $C={\left(\left(s_{22}+\lambda_{22}\right)\Omega_{11}^{-1}+\text{diag}\left(\tau_{12}\right)\right)}^{-1}$,
  compute **ω**_**12**_ = ***β***, $\omega_{22}=\gamma +\beta^{T}\Omega_{11}^{-1}\beta$;
7. Rescale $\tilde{\Psi }$ and $\tilde{\Gamma }$
$\tilde{\Gamma }=\Omega^{-1}$,
$\Gamma_{\left[gh\right]}={\tilde{\Gamma }}_{\left[gh\right]}/\sqrt{\left({\tilde{\Gamma }}_{\left[gg\right]}+{\tilde{\Psi }}_{\left[gg\right]}\right)\left({\tilde{\Gamma }}_{\left[gg\right]}+{\tilde{\Psi }}_{\left[gg\right]}\right)}$,
$\Psi_{\left[gh\right]}={\tilde{\Psi }}_{\left[gh\right]}/\sqrt{\left({\tilde{\Gamma }}_{\left[gg\right]}+{\tilde{\Psi }}_{\left[gg\right]}\right)\left({\tilde{\Gamma }}_{\left[gg\right]}+{\tilde{\Psi }}_{\left[gg\right]}\right)},g,h=1,...,p$;
8. Sample the latent variables *z*_*icl*_, *c* = 1, …, *m*, *i* = 1, …, *n*_*c*_, *l* = 1, …, *p*
$z_{icl}∼\text{TN}\left(b_{cl}+\Gamma_{l,-l}\Gamma_{-l,-l}^{-1}\left(z_{\text{ic},-l}-b_{c,-l}\right),\;\Gamma_{l,-l}\Gamma_{-l,-l}^{-1}\Gamma_{-l,l}\right)$, which is a truncated normal distribution with lower bound as *lb* = *max*(*z*_*hl*_: *y*_*hl*_ < *y*_*icl*_) and upper bound as *ub* = *min*(*z*_*hl*_: *y*_*hl*_ > *y*_*icl*_), *h* = 1…, *N*. We use **z**_**ic**,**−l**_ to denote the vector of **z** without variable *l*, **Γ**_**l**,**−l**_ denotes the *l*^*th*^ row of **Γ** matrix without the *l*^*th*^ column, and **Γ**_**−l**,**−l**_ denotes **Γ** matrix without the *l*^*th*^ row and the *l*^*th*^ column.

### 5.2 Appendix B: Simulation study on choosing hyper-parameter

We designed a simple simulation study to illustrate the effect of choosing a different hyper-parameter *t* on parameter estimation. The number of clusters was 20, and the cluster sizes were unbalanced, and generated from a Poisson distribution with mean 50. We generated 100 replicate data sets with the true parameters as

$$\Psi =(\begin{array}{cccc} 0.562 & 0.090 & 0.140 & -0.028\\ 0.090 & 0.212 & 0.053 & 0.030\\ 0.140 & 0.053 & 0.379 & 0.050\\ -0.028 & 0.030 & 0.050 & 0.505 \end{array}),$$

$$\Omega =(\begin{array}{cccc} 3.183 & -0.918 & 0.067 & 1.209\\ -0.918 & 1.545 & -0.028 & -0.498\\ 0.067 & -0.028 & 1.613 & 0.046\\ 1.209 & -0.498 & 0.046 & 2.497 \end{array}).$$

The hyper-parameter *s* was fixed as 0.01, and *t* varied to be 10^−1^, 10^−3^ and 10^−5^ respectively. We calculated the 95% highest density region of each parameter from the MCMC in each data set to see if the true parameter was included in the region, and then computed the coverage over the 100 data sets. The procedure was repeated with each of the three *t* values.

**Fig 3:**
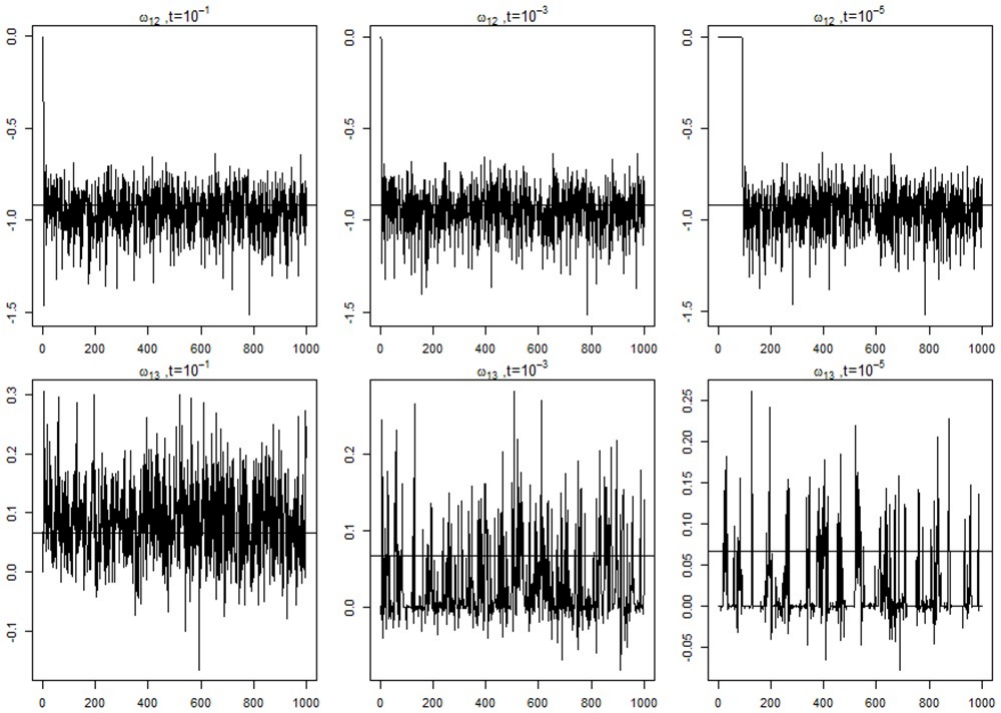
Graphic representation of conditional dependence among the questions in the MDOT data set. Edges in red denote positive relationships and blue denote negative relationships. Questions from different sections in the questionnaire are plotted with different colors.

The coverage rates of the unique elements in the **Ψ** and **Ω** matrices are reported in Table 4. The coverage rates were generally close to the nominal 95% level in all the parameters in the *Ψ* matrix for the random effects regardless of different *t* values. This is because we did not shrink on the **Ψ** matrix. For the **Ω** matrix, it is noticed that larger parameters were insensitive to *t* values whereas smaller parameters suffered from under coverage with small *t* values. To see the different shrinkage effects of *t* more clearly, we show the trace plots of two selected parameters in **Ω** with the three *t* values in Fig 3. In the upper panel the true parameter *ω*_12_ = −0.918 was relatively large, and the MCMC samples were centered around the true parameter with all the different *t* values. In the lower panel when the true parameter *ω*_13_ = 0.067 was relatively small, the model with *t* = 10^−1^ achieved 96% coverage rate and did not show much shrinkage effect, however the models with *t* = 10^−3^ and *t* = 10^−5^ shrunk *ω*_13_ towards 0 and their coverage rates were down to 89% and 63% respectively. Although the coverage rates were lower than 95% for small parameters when *t* was small, it is desirable to see that the model was able to filter out the parameters with weaker associations, and retain the stronger ones.

**Table 4:** Coverage rates of the unique parameters in the Ω and Ψ matrices.

Ω	True parameters	Coverage (%)			Ψ	True parameters	Coverage (%)		
		t = 10^−1^	t = 10^−3^	t = 10^−5^			t = 10^−1^	t = 10^−3^	t = 10^−5^
*ω*_11_	3.183	90	91	90	*ψ*_11_	0.562	91	91	90
*ω*_12_	-0.918	97	97	96	*ψ*_12_	0.090	94	95	95
*ω*_13_	0.067	96	89	63	*ψ*_13_	0.140	92	93	92
*ω*_14_	1.209	91	89	89	*ψ*_14_	-0.028	94	93	93
*ω*_22_	1.545	91	87	87	*ψ*_22_	0.212	95	95	95
*ω*_23_	-0.028	97	89	84	*ψ*_23_	0.053	96	96	96
*ω*_24_	-0.498	90	94	93	*ψ*_24_	0.030	98	98	96
*ω*_33_	1.613	93	93	91	*ψ*_33_	0.379	98	99	98
*ω*_34_	0.046	97	97	75	*ψ*_34_	0.050	97	98	98
*ω*_44_	2.497	89	89	88	*ψ*_44_	0.505	96	97	97

## Supporting information

S1 File (PDF)

## References

[1] Suvitie P, Hallamaa M, Matomäki J, Mäkinen J, Perheentupa A. Prevalence of pain symptoms suggestive of endometriosis among finnish adolescent girls (TEENMAPS study). Journal of pediatric and adolescent gynecology. 2016;29(2):97–103. 10.1016/j.jpag.2015.07.001 26169662

[2] Esen İ, Oğuz B, Serin H. Menstrual characteristics of pubertal girls: a questionnaire-based study in Turkey. Journal of clinical research in pediatric endocrinology. 2016;8(2):192. 10.4274/jcrpe.2026 26758209 PMC5096475

[3] De Sanctis V, Soliman A, Elsedfy H, Soliman N, Soliman R, El Kholy M. Dysmenorrhea in adolescents and young adults: a review in different country. Acta Bio Medica Atenei Parmensis. 2017;87(3):233–246. PMC10521891 28112688

[4] Agarwal A, Venkat A. Questionnaire study on menstrual disorders in adolescent girls in Singapore. Journal of pediatric and adolescent gynecology. 2009;22(6), pp.365–371. 10.1016/j.jpag.2009.02.005 19647453

[5] Overton C, Hargreaves J, Maresh M. A national survey of the complications of endometrial destruction for menstrual disorders: the MISTLETOE study BJOG: An International Journal of Obstetrics Gynaecology. 1997; 104(12), pp.1351–1359. 10.1111/j.1471-0528.1997.tb11003.x 9422012

[6] Cakir M, Mungan I, Karakas T, Girisken I. Menstrual pattern and common menstrual disorders among university students in Turkey. Pediatrics International. 2007;49(6), pp.938–942. 10.1111/j.1442-200X.2007.02489.x 18045301

[7] Parker M, Sneddon A, Arbon P. The menstrual disorder of teenagers (MDOT) study: determining typical menstrual patterns and menstrual disturbance in a large population-based study of Australian teenagers. BJOG: An International Journal of Obstetrics & Gynaecology. 2010;117(2):185–192. 10.1111/j.1471-0528.2009.02407.x 19874294

[8] Tibshirani R. Regression shrinkage and selection via the lasso. Journal of the Royal Statistical Society: Series B (Methodological). 1996;58(1):267–288.

[9] Rue H, Held L. Gaussian Markov random fields: theory and applications. CRC press; 2005.

[10] Nelsen R. An introduction to copulas. Springer Science & Business Media; 2007.

[11] Hoff P. Extending the rank likelihood for semiparametric copula estimation. The Annals of Applied Statistics. 2007;p. 265–283. 10.1214/07-AOAS107

[12] Yuan M, Lin Y. Model selection and estimation in the Gaussian graphical model. Biometrika. 2007;94(1):19–35. 10.1093/biomet/asm018

[13] Friedman J, Hastie T, Tibshirani R. Sparse inverse covariance estimation with the graphical lasso. Biostatistics. 2008;9(3):432–441. 10.1093/biostatistics/kxm045 18079126 PMC3019769

[14] Mohammadi A, Wit E. Bayesian structure learning in sparse Gaussian graphical models. Bayesian Analysis. 2015;10(1):109–138. 10.1214/14-BA889

[15] Marlin B, Schmidt M, Murphy K. Group sparse priors for covariance estimation. In: Proceedings of the Twenty-Fifth Conference on Uncertainty in Artificial Intelligence. AUAI Press; 2009. p. 383–392.

[16] Lenkoski A, Dobra A. Computational aspects related to inference in Gaussian graphical models with the G-Wishart prior. Journal of Computational and Graphical Statistics. 2011;20(1):140–157. 10.1198/jcgs.2010.08181

[17] Wang H. Bayesian graphical lasso models and efficient posterior computation. Bayesian Analysis. 2012;7(4):867–886. 10.1214/12-BA729

[18] Wang J, Loong B, Westveld A, Welsh A. A Copula-based Imputation Model for Missing Data of Mixed Type in Multilevel Data Sets. arXiv preprint arXiv:170208148. 2017;.

[19] Fan J, Feng Y, Wu Y. Network exploration via the adaptive LASSO and SCAD penalties. The annals of applied statistics. 2009;3(2):521. 10.1214/08-AOAS215 21643444 PMC3105782

[20] Genest C, Ghoudi K, Rivest L. A semiparametric estimation procedure of dependence parameters in multivariate families of distributions. Biometrika. 1995;82(3):543–552. 10.1093/biomet/82.3.543

[21] Mohammadi A, Abegaz F, Heuvel E, Wit E. Bayesian modelling of Dupuytren disease by using Gaussian copula graphical models. Journal of the Royal Statistical Society: Series C (Applied Statistics). 2017;66(3):629–645.

[22] Gelman A, Stern H, Carlin J, Dunson D, Vehtari A, Rubin D. Bayesian data analysis. Chapman and Hall/CRC; 2013.

[23] Mohammadi A, Wit E. BDgraph: an R package for Bayesian structure learning in graphical models. arXiv preprint arXiv:150105108. 2015;.

[24] Csardi G, Nepusz T. The igraph software package for complex network research. InterJournal, Complex Systems. 2006;1695(5):1–9.

[25] Arruda M, Petta C, Abrao M, Benetti-Pinto C. Time elapsed from onset of symptoms to diagnosis of endometriosis in a cohort study of Brazilian women. Human Reproduction. 2003;18(4):756–759. 10.1093/humrep/deg136 12660267

[26] Dmowski W, Lesniewicz R, Rana N, Pepping P, Noursalehi M. Changing trends in the diagnosis of endometriosis: a comparative study of women with pelvic endometriosis presenting with chronic pelvic pain or infertility. Fertility and Sterility. 1997;67(2):238–243. 10.1016/S0015-0282(97)81904-8 9022596

[27] Albert J, Chib S. Bayesian analysis of binary and polychotomous response data. Journal of the American Statistical Association. 1993;88(422):669–679. 10.1080/01621459.1993.10476321

[28] Yuan M, Lin Y. Model selection and estimation in regression with grouped variables. Journal of the Royal Statistical Society: Series B (Statistical Methodology). 2006;68(1):49–67. 10.1111/j.1467-9868.2005.00532.x

[29] Kyung M, Gill J, Ghosh M, Casella G. Penalized regression, standard errors, and Bayesian lassos. Bayesian Analysis. 2010;5(2):369–411. 10.1214/10-BA607

