# Supplementary material for: Analysis of survey on menstrual disorder among teenagers using Gaussian copula model with graphical lasso prior
Source: PLoS One. 2021 Mar 18;16(3):e0248340. doi: 10.1371/journal.pone.0248340 (PMC7971494; doi:10.1371/journal.pone.0248340)
Supplement: S1 File — (PDF) [file pone.0248340.s001.pdf]

Please note that this questionnaire is 6 pages long, and has a glossary of unfamiliar words and terms at the end to help you.

Section 1. General information

Today's date: _______/______/______ (dd/mm/yy) Weight:_________kgs (approx)
Your DOB: _______/______/______ (dd/mm/yy) Height:_________cms (approx)
How old were you when you got your first period? _______yrs (approx)

Section 2. About your usual periods (*Please circle the appropriate response*)

1. Do you have periods? Yes No (If no, go to Section 5)

2. Over the past 12 months have your periods been:
regular irregular don't know
(regular = the time between periods is usually about the same length, irregular = if the length of time between periods often changes)

3. What is the ***usual*** number of days from the first day of bleeding at one period to the first day of bleeding at your next period? (Cycle length)
__________days periods irregular don't know

4. Please tick the column that indicates the heaviness of your bleeding for each day of your period (how it usually is):

Day	Light	Medium	Heavy
1			
2			
3			
4			
5			
6			
7			
8			
9			
10			

If longer, please specify how many more days: _______________________________

5. Does your bleeding contain clots?
Yes No (if no, go to question 7)

6. If 'yes', how often does it contain clots?
Sometimes Most of the time All the time

7. Do you ever notice spots of blood on your underpants?
Just before a period In between periods Never

8. Do you miss school because of your periods?
No……(please go to question 11)
Yes – Every period
Yes – Just with some periods

9. If yes, how many days of your period do you usually stay home for? _____________

10. What is it about your period that causes you to miss school? (You can circle more than one)
Too painful Blood flow too heavy Nausea Vomiting Other______________

11. Have your period symptoms (such as the ones in question 10) worsened over the past 12 months?
Yes No Not Applicable

© Melissa Parker 2004

12. Please rate any period pain you have had over the past 6 months?<br>No pain 0 1 2 3 4 5 6 7 8 9 10 Worst pain<br>(If 'no pain', please go to Section 3)
13. If you have period pain do you take medication?<br>Yes No (If no, please go to Section 3)
14. If yes, which medication/s do you usually take? (You can circle more than one)<br>Panadol Aspirin/Aspro Ponstan Naprosyn Nurofen<br>Other/s ________________
14. How effective is the medication in relieving your pain?<br>Not effective 0 1 2 3 4 5 6 7 8 9 10 Highly effective

<u>Section 3</u>. Over the past 12 months, have you experienced any of the following symptoms in relation to your monthly period cycle? (*Tick as many as applicable*)

		Doesn't apply to me	No or Never	Just before a period	At the time of period	Any time of the month	All the time	Sometimes
Nausea (feel like vomiting)								
Vomiting								
Bloating (swollen tummy)								
Diarrhoea/constipation or both								
Indigestion, reflux, heartburn								
Changes in appetite								
Aching outside your vagina								
Aching down the legs								
Pelvic pain	aching							
	cramping							
	stabbing							
	Other (please state):							
Lower back pain								
Pain during or after passing urine								
Pain when your bladder is full								
Pain before or when passing wind								
Pain when emptying your bowels								
Feeling an urgent need to empty your bowels								
Bleeding from your bottom (anus)								

© Melissa Parker 2004

	Doesn't apply to me	No or Never	Just before a period	At the time of period	Any time of the month	All the time	Sometimes
Pain during or after sexual intercourse							
Need to pass urine often							
Feeling really tired							
Headaches							
Thrush (itchy and sore around and outside the vagina)							
Dizziness, fainting or passing out							
Feeling 'down' or depressed							
Other symptoms:_____________________________							

Section 4.

Do your periods affect your lifestyle?

Please circle a number between 0 and 10 to show us how much your periods *over the past 12 months* have interfered with the following:

0 = no interference, 10 = major interference, N/A = Not Applicable (to me)

Activity	NO INTERFERENCE	Scale of 0 to 10											MAJOR INTERFERENCE	N/A
Attending school		0	1	2	3	4	5	6	7	8	9	10		
Completing school work		0	1	2	3	4	5	6	7	8	9	10		
Casual paid work		0	1	2	3	4	5	6	7	8	9	10		
Social activities		0	1	2	3	4	5	6	7	8	9	10		
Relationship with family		0	1	2	3	4	5	6	7	8	9	10		
Relationship with friends		0	1	2	3	4	5	6	7	8	9	10		
Relationship with partner		0	1	2	3	4	5	6	7	8	9	10		
Sexual activity		0	1	2	3	4	5	6	7	8	9	10		
Sport and exercise		0	1	2	3	4	5	6	7	8	9	10		

Does the above interference with lifestyle occur with:

Some periods   Most periods   All periods   Not applicable to me

© Melissa Parker 2004

What is it about your period that interferes with your life?

Please circle a number between 0 and 10 to show us how much the following symptoms interfere with your life.

() = no interference, 10 = major interference, N/A = Not Applicable (to me)

Symptom		Scale of 0 to 10												N/A
Pain	NO INTERFERENCE	0	1	2	3	4	5	6	7	8	9	10	MAJOR INTERFERENCE	
Heavy blood flow		0	1	2	3	4	5	6	7	8	9	10		
Tiredness/fatigue		0	1	2	3	4	5	6	7	8	9	10		
Moods		0	1	2	3	4	5	6	7	8	9	10		
Generally feeling unwell		0	1	2	3	4	5	6	7	8	9	10		
Other_________________ (Please name symptom)		0	1	2	3	4	5	6	7	8	9	10		

Does the above interference occur with:

Some periods Most periods All periods Not applicable to me

<u>Section 5</u>. The following is a list of statements related to periods. Please place a tick in the column that best represents your own experience for each statement.

N/A = Not Applicable (to me)

	True	False	Don't know	N/A
I usually have a period every month				
I have never missed a period				
I have had problems with my periods				
My periods seem pretty normal				
I have had tests because things weren't right with my period				
I have a period problem that has a name: (Please state)____				
I am on the pill				
I take the pill to regulate my periods				
I take the pill to prevent pregnancy				
I take the pill to help period pain				
I have never taken the pill				
Periods don't worry me too much				
Periods worry me a lot				

© Melissa Parker 2004

		True	False	Don't know	N/A
I think my periods are 'normal' most of the time					
I sometimes think there is something 'wrong' with my periods					
I am sure there is something 'wrong' with my periods					
I have never used a tampon					
I have tried to use a tampon but can't get it in					
I am not interested in using a tampon					
I can insert a tampon but it is too uncomfortable					
I only use tampons					
I only use pads (sanitary napkins)					
I use pads and tampons					
I often get lots of pimples on my	Face				
	Back				
	Chest				
I have more hair than usual growing on my body					
I've had a blood test for my period pain					
I've had an ultrasound to look for causes of my pain					
I've had an operation to look for causes of my pain					
I talk to my friends about my periods					
I talk to someone in my family about my periods					
I've talked to my teacher/school counselor about my periods					
I've talked to my GP about my periods					
I've talked to a specialist doctor about my periods					
I've talked to a naturopath/herbalist/acupuncturist about my periods					
I am grumpy before or during my periods					
I am grumpy all the time					
I get teary before or during a period					
I feel overwhelmed and not able to cope before or during a period					
I often want to withdraw or hide when I have my period					
My periods don't affect my moods					

Section 6. Please list any allergies or intolerances you have to the following:

Medication/s:
Foods:
Other (eg bee stings):

© Melissa Parker 2004

Section 7.

Have you ever heard of any of the following?	Yes	No	Don't know
Polycystic ovarian syndrome/polycystic ovaries (PCOS)			
Endometriosis			
Pelvic inflammatory disease (PID)			

Have you ever been diagnosed with of any of the following?	Yes	No	Don't know
Polycystic ovarian syndrome/polycystic ovaries (PCOS)			
Pelvic inflammatory disease (PID)			
Endometriosis			
If 'yes' for endometriosis, was diagnosis based on symptoms?			
If 'yes' for endometriosis, was diagnosis made after an operation (Laparoscopy) to look inside your abdomen?			

Does your mother or sister have any of the following?	Yes	No	Don't know	N/A
Period problems				
Severe period pain				
Pelvic inflammatory disease				
Polycystic ovarian syndrome/polycystic ovaries				
Endometriosis				

Is there anything else that you would like to tell us about your periods or something that has changed your periods? (Any extra information is helpful for the researchers)

____

____

____

You have finished! Thank you for completing this questionnaire.

Glossary If there are other words in the questionnaire that you do not understand please raise your hand and someone will help you.

Word/terms	Meaning
emptying bowels:	doing a poo
indigestion:	heartburn, stomach pain after eating, reflux, acid pain in the stomach
pelvic pain:	pain in lower tummy, below belly button
passing wind:	farting
diagnosis	the identification of an illness (usually by a doctor/health professional)
Not Applicable or N/A:	this doesn't apply to me

© Melissa Parker 2004

© Melissa Parker 2004
